# Testing for associations between systolic blood pressure and single-nucleotide polymorphism profiles obtained from sparse principal component analysis

**DOI:** 10.1186/1753-6561-8-S1-S95

**Published:** 2014-06-17

**Authors:** Ashley Bonner, Binod Neupane, Joseph Beyene

**Affiliations:** 1 Clinical Epidemiology and Biostatistics Department, McMaster University, Hamilton, Ontario, Canada L8S 4L8

## Abstract

**Background: **Hypertension is a prevalent condition linked to major cardiovascular conditions and multiple other comorbidities. Genetic information can offer a deeper understanding about susceptibility and the underlying disease mechanisms. The Genetic Analysis Workshop 18 (GAW18) provides abundant genotype data to determine genetic associations for being hypertensive and for the underlying trait of systolic blood pressure (SBP). The high-dimensional nature of this data promotes dimension reduction techniques to remove excess noise and also synthesize genetic information for complex, polygenic traits. **Methods: **For both measured and simulated phenotype data from GAW18, we use sparse principal component analysis to obtain sparse genetic profiles that represent the underlying data structures. We then detect associations between the obtained sparse principal components (PCs) and SBP, a major indicator of hypertension, following up by investigating the sparse PCs for genetic structure to gain insight into new patterns. **Results: **After adjusting for multiple testing, 27 of 122 PCs were significantly associated with measured SBP, offering a large number of components to investigate. Considering the top 3 PCs, linked genetic regions have been identified; these may act in unison while associated with SBP. Simulated data offered similar results. **Conclusions: **Sparse PCs can offer a new data-driven approach to structuring genotype data and understanding the genetic mechanics behind complex, polygenic traits such as hypertension.

## Background

Hypertension is a condition linked to major cardiovascular issues that result in heart failure and increased death rates [1-3]. Many risk factors, such as obesity, aging, and smoking, have been attributed to an individual's susceptibility to hypertension, but recent access to abundant genetic data has shifted investigation in a new and exciting direction. The Genetic Analysis Workshop 18 (GAW18) exemplifies not only this transition of focus but also the realization of new data-related challenges.

Offering genotype data for more than 24 million single-nucleotide polymorphisms (SNPs) on odd-numbered autosomal chromosomes from 959 individuals, the goal of GAW18 is to determine genetic markers that play a crucial role in blood pressure levels. A data environment of this size is termed high-dimensional, referring to the vast number of variables, and poses many statistical challenges relating to insufficient degrees of freedom when modeling (because *n < p*) and multiple testing; it creates an interesting platform to practice new statistical tools that confront these challenges. In particular, dimension-reduction methods that narrow our focus to the important data features could be extremely beneficial.

Principal component analysis (PCA) is a commonly used multivariate method for both dimension reduction and data visualization. It defines a new and convenient set of variables Z, called *principal components *(PCs), as linear combinations of original variables X. These PCs are uncorrelated and ordered by maximal variance, possibly giving the analyst an easier data set to work with. If most of the original variance is held by the first few PCs, one can discard the rest and work with a much lower dimensional problem without colinearity. However, making sense of what the PCs represent is often an issue because they are linear combinations of *all *original variables. To alleviate this limitation, *sparse *PCA methods [5-8] have been developed by incorporating methodology related to penalized regression [4] to achieve sparse coefficient, or *loading*, vectors. *Tuning parameters *control the level of sparseness induced, making the procedures very flexible. Setting most of the loadings to exactly 0, PCs can now be tied to tight groups of original variables that remain, resulting in an interpretable version of classical PCA.

In this paper, we use sparse PCA methodology to inform group structure in a portion of the GAW18 single-nucleotide polymorphism (SNP) data before moving on to model systolic blood pressure (SBP), a surrogate of hypertension, with representative sparse PCs. Through interpreting the PCs significantly associated with SBP, it is our goal to reveal unique data-driven groups of SNPs associated with our phenotype of interest. Because SBP is a polygenic trait, we expect the PCs to shed new light on structures of genetic regions that might act together. After describing our choice of data and methods of analysis, we will present our results and some discussion. We end by summarizing the limitations with using sparse PCA to handle genotype data and discussing future ideas.

## Methods

### Data description

The GAW18 data providers obtained real phenotype, covariate, and genotype data from 959 possibly related individuals. Fixing the genotype data, they prescribed a model-based relationship between functional genotypic regions, informed by the real data set and external sources, to simulate 200 phenotype-covariate data sets. We will use the real phenotype data to demonstrate an uninformed analysis that would occur in practice and use the simulated phenotype data to check the ability of our analysis to consistently detect similar group structure surrounding some truly associated SNPs.

*Target sample*. Of the 959 individuals from whom data is available, we chose to use only the 157 unrelated individuals.

*Target variables (phenotype and covariates)*. Although the GAW18 group provided longitudinal phenotype and covariate data, we focus on just the baseline measures. We chose SBP as our target phenotype variable because its continuous structure allows detection of modest changes. We adjust for age, sex, smoking status, and use of blood pressure medication as covariates when attempting to detect associations between SBP and genotype information.

*Target variables (genotype)*. The genotype data provided contained more than 24 million SNPs found on odd-numbered autosomes. Following the GAW18 group suggestions in the guide, we aim at chromosome 3. Furthermore, we consider only the 65,519 SNPs from the genome-wide association studies (GWAS) file of chromosome 3 because the sparse PCA method requires a sufficient level of variation.

*Final data*. There were instances of missing data in the 157 unrelated subjects and 65,519 SNPs. After removing individuals with missing phenotype or covariate data, we used PLINK software to remove individuals with genotypic calling error rates greater than 5% and remove SNPs that had missing data, minor allele frequency (MAF) less than 5%, or that failed the Hardy-Weinberg equilibrium (HWE) test at 0.001 level of significance. The final real phenotype data set we work with has 122 unrelated individuals with complete data for SBP, age, sex, smoking status, hypertension medication, and 46,574 SNPs. The final 200 simulated phenotype data sets each have complete data on 133 individuals because there was no missing data in phenotypes or covariates.

### Analysis description

We used a 3-step process to ultimately determine groups of SNPs jointly related to SBP for the real phenotype data and each of the 200 simulated phenotype data sets. An additive model for SNPs was assumed, meaning SNPs (coded 0, 1, and 2 for copies of the minor allele) were taken as continuous.

*Step 1: SNP-by-SNP filtering*. To reduce the computational burden for our sparse PCA method, we first applied a linear regression model for each SNP individually, retaining those that were statistically associated with SBP at a 5% level of significance after adjusting for age, sex, smoking status, and blood pressure medication covariates. This also ensures our sparse PCA method will not accept marginally insignificant SNPs that could draw attention away from those that are most likely to be of effect. We did not adjust for multiple testing here because this step is merely used as a filter.

*Step 2: Sparse PCA to obtain groups*. Taking SNPs retained from step 1, we performed the sparse PCA method published in 2009 by Witten et al [6] to obtain a less dimensional yet representative set of PCs. The remaining nonzero loadings of these sparse PCs correspond to the SNPs contributing to their linear combinations. Prior work suggests this sparse PCA method could be the best choice among competing sparse PCA methodology when facing such a high-dimensional (*n < p*) data situation [8]. *Tuning parameter selection: *From the real phenotype data set, we selected a tuning parameter that maintained a balance between adjusted-percentage-explained variance of PCs as calculated by Witten et al [6] and sparseness of loading vectors among the sparse PCs. This tuning parameter was then used for each of the 200 simulated data sets to keep dimension reduction consistent.

*Step 3: Relating sparse PCs to SBP*. Finally, we applied a linear regression model for each sparse PC individually, retaining those that were topmost statistically associated with SBP. We then cross-referenced the SNPs with nonzero loadings in these significant sparse PCs with gene information to attribute genetic structure to the groupings and see if new structures emerged. We report the most prevalent genetic regions.

Alongside these steps, we also tracked progress of two of the topmost contributing SNPs in the underlying simulation model. They were rs6442089 from the *MAP4 *gene (β = −2.3810) and rs1131356 from the *FLNB *gene (β = 1.0007). We were restricted to following these because they were the only notable contributing SNPs still remaining in our GWAS chromosome 3 data; rare variants (i.e., SNPs with MAF <5%) were given heavier weights in the simulation model.

All steps were performed within the statistical programming language R (version 2.15.2). To apply the sparse PCA method, we used the authors' published R-package called PMA [6].

## Results

### Real phenotype data analysis

*Step 1*. Of the 46,574 SNPs in our real data, 2256 were deemed to be statistically associated with SBP at a 5% level of significance. Minimum *p*-value was 1.72 × 10^-5^. Therefore, we retained these SNPs into the sparse PCA phase with a data set of 122 individuals and 2256 SNPs.

*Step 2*. Tuning parameter selection must be appropriate because it decides how sparse the obtained PCs are at the expense of adjusted-percentage-explained variance. Attempting tuning parameters λ = 3, 4, 5, 6, 7, 8, 9, 10, 12, 14, 16, 18, 20, 25, 30, we gauged that choosing tuning parameter λ = 10 resulted in a balance between adjusted-percentage-explained variance and sparseness. This was graphically determined based on diminishing returns in both criteria across tuning parameters. Performing the sparse PCA, a mean (SD) of 246.2 (44.1) nonzero loadings per PC existed across the 122 PCs, reflecting that a decent level of sparseness was introduced to the PCA procedure. These PCs retained 66.1% of the total variance across *p *= 2256 SNPs that entered the sparse PCA phase. This is a relatively large adjusted-percentage-explained variance because roughly 90% of the loadings were forced to 0.

**Figure 1:**
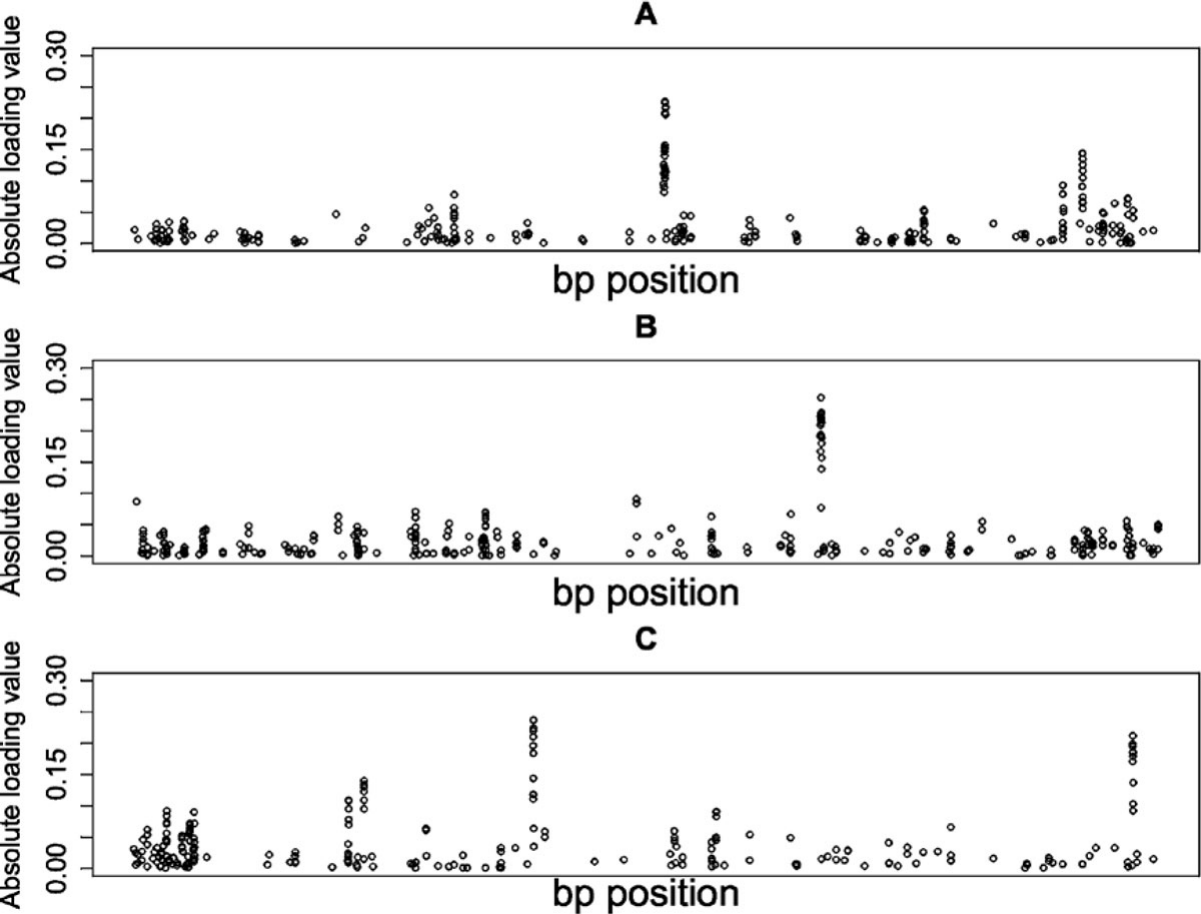
**Visualizing single-nucleotide polymorphism profiles from the top 3 sparse principal components (PCs) associated with systolic blood pressure**. (A) Sparse PC 11. (B) Sparse PC 9. (C) Sparse PC 19. Plots of the loading values for a PC to visually inspect genetic structure. bp, base-pair.

*Step 3*. Of the 122 sparse PCs obtained from the real data, 27 were deemed statistically associated with SBP at a Bonferroni-corrected 5/122 = 0.00041% level of significance; because PCs tend to be uncorrelated, the Bonferroni correction here is appropriately conservative. The minimum *p*-value was 4.37 × 10^-8^. Figure 1 displays plots of the loadings from the top 3 PCs that are statistically associated with SBP (PC11, PC9, and PC19). The SNP base-pair positions are plotted along the x-axis, and the absolute loading values (weights) are plotted along the y-axis, giving an intuitive look at SNP profiles based on their contribution to the significantly associated PC. Genetic regions that are prevalent and hold large weights will display as a tall peak on the graph. In PC11, the largest loading values occur along the intergenic region between *ZPLD1 *and *MIR548A3 *genes and within the *LAMP3 *and *MCCC1 *gene. In PC9, the largest loading values were found along the intergenic region between *NPHP3-AS1 *and *TMEM108 *genes, as well as within the *TMEM108 *gene. In PC19, the largest loadings occurred within the *ROBO2 *gene, along the intergenic region between *CDCP1 *and *TMEM158 *genes, within the *FGF12 *gene, and along the intergenic region between *FGF12 *and *MD21D2*. Table 1 provides the number of SNPs that contribute to each PC from these genetic regions, as well as the percentage of contribution.

**Table 1:** Notable genetic regions from top 3 sparse principal components associated with systolic blood pressure

PC 11			PC 9			PC 19		
		
Genetic region^1^	# SNPs in PC	**% Load contrib**.	Genetic region	# SNPs in PC	**% Load contrib**.	Genetic region	# SNPs in PC	**% Load contrib**.
*ZPLD1 *to *MIR548A3*	28	0.686748	*NPHP3-AS1 *to *TMEM108*	13	0.563824	*ROBO2*	11	0.336118
*LAMP3*	7	0.071419	*TMEM108*	6	0.209901	*FGF12*	5	0.151836
*MCCC1*	4	0.070489				*FGF12 *to *MB21D2*	5	0.119935
						*CDCP1 *to *TMEM158*	6	0.090491

^1^Genetic region is either the gene symbol or intergenic region (e.g., gene1 to gene2).

*PC*, principal component; *SNP*, single-nucleotide polymorphism.

### Simulated phenotype data analysis

*Step 1*. Of the 46,574 SNPs in each simulated data set, a mean (SD) of 2385.4 (232.0) across the 200 data sets was deemed statistically associated with SBP at a 5% significance. Minimum *p*-values had a mean (SD) of 3.65 × 10^-5 ^(3.79 × 10^-5^); rs6442089 from the *MAP4 *gene was deemed significant in 38 of 200 simulated data sets, and rs1131356 from the *FLNB *gene was deemed significant in only 8 of the 200 simulated data sets. Because we would expect 10 out of 200 samples to result in a false positive if there were no underlying association (i.e., if β = 0), rs6442089 (β = −2.3810) has been detected an appropriately increased number of times, but rs1131356 (β = 1.0007) seems unrelated. For this reason, we now look exclusively at rs6442089.

*Step 2*. Because our focus is to track a specific SNP, we will follow only those data sets that contain rs6442089. Tuning parameters were fixed at λ = 10 to keep sparseness and adjusted-percentage-explained variance consistent with the real phenotype data analysis. For each of the 38 data sets that retained rs6442089, we ran sparse PCA to obtain the 133 sparse PCs.

*Step 3*. Of the 133 sparse PCs obtained for each of the 38 data sets that retained rs6442089, a mean (SD) of 28.1 (8.5) PCs was deemed statistically associated with SBP at a Bonferroni-corrected 5/133 = 0.00038% level of significance. From each of the 38 lists of significant PCs, we then identified the PC for which rs6442089 had the largest contribution (absolute loading value) and detected genetic structure from the SNPs (loadings) in this PC. Among the 38 PCs chosen in this way, the *MAP4 *gene had a large set of SNPs with substantial loading contribution; this is as expected because rs6442089 is from *MAP4*. With few exceptions, the *SMARCC1 *gene was equivalent in both SNP frequency and loading contribution, suggesting it may be linked with *MAP4*. This consistency shows the robustness of sparse PCA to noise generated in the phenotype; likewise, but less consistent and impactful, were the *DHX30 *and *CSPG5 *genes. Intergenic regions between *CSPG5 *and *SMARCC1, SMARCC1 *and *MAP4*, and *MAP4 *and *CDC25A *were also notable.

## Discussion

The sparse PCs constructed from a list of marginally associated SNPs gives insight into a new grouping structure. With a genetic component that is complex and polygenic, understanding the genetics behind SBP from this data-driven angle may be rewarding. Perhaps genes and intergenic regions that are highly weighted within the same PC have some functional similarities, or act in unison.

The choice of tuning parameter heavily influences our conclusions. We could have, for example, chosen a tuning parameter such that there were 10 to 20 remaining nonzero loadings for each loading vector. This would allow us to focus on the last groups of SNPs remaining, but with drastically increased sparseness comes loss of information, namely, severe reduction in adjusted-percentage-explained variance and loss of the SNPs of interest for the simulated data. Prior biological understanding, including specifying candidate genetic regions, could help to guide decisions regarding the tuning parameter.

The strategy we followed in this paper has some limitations. For example, concentrating only on unrelated individuals reduced our sample size, leading to low power when working with the simulated data, and limited our ability to detect the two SNPs of interest. Similarly, because we chose to focus on the GWAS file, containing only common SNPs (MAF >5%), we were not able to search for the rare-variant SNPs with higher coefficient weights in the underlying simulation model. Step 1 of our analysis strategy informs, or supervises, our sparse PCA. We could have discarded step 1 in favor of unsupervised sparse PCA to search for SNPs jointly associated with SBP, although not marginally; any finding here could be very interesting. Our assumptions regarding the genotype data structure may have influenced the results from our analysis. Considering each SNP as continuous is usually a necessity when applying sparse PCA; however, this assumes an additive model from which only linear effects can be measured.

It would be interesting to investigate performance of sparse PCA using the variance-covariance matrix with the similarity measure developed and explained by Niitsuma and Okada (2005) that is meant to handle categorical data such as SNPs [9,10]. Also, investigating differences in findings between our supervised approach and an unsupervised approach to sparse PCA could be an excellent simulation study for future work.

## Conclusions

As we have demonstrated, sparse PCA methodology is able to reduce the dimension of SNPs and reveal groups potentially related to a phenotype of interest. It can also be applied to many other, perhaps more suitable, data types and could be of significant benefit to researchers attempting to handle high-dimensional data, especially when considering complex, polygenic traits.

## Competing interests

The authors declare that they have no competing interests.

## Authors' contributions

AB designed the overall study, conducted statistical analyses, and drafted the manuscript; BN assisted with statistical analyses and interpretation; and JB conceived the overall study and assisted in drafting and critical revision of the manuscript. All authors read and approved the final manuscript.
